# Advance Directives & Unlimited Treatment Preference in Dementia Scenario: Insights of Community-Dwelling Adults

**DOI:** 10.1093/geroni/igaf122.2915

**Published:** 2025-12-31

**Authors:** Yuchi Young, Yichun Liu, Yufang Tu, Wan-Yu Chiu, Ashley Shayya, Thomas O’Grady

**Affiliations:** University at Albany College of Integrated Health Sciences, Albany, New York, United States; New York-Presbyterian Hospital, New York, New York, United States; North Dakota State University, Fargo, North Dakota, United States; National Taiwan University, Zhongzheng District, Taipei, Taiwan (Republic of China); Center for Human Services Research, University at Albany College of Integrated Health Sciences, Albany, New York, United States; University at Albany, Albany, New York, United States

## Abstract

Dementia leads to progressive cognitive decline, impairing self-care and decision-making. Advance directives (AdvDirs) enable individuals to document healthcare preferences while cognitively capable, ensuring value-aligned care and reducing caregiver burden. This study explores factors influencing preferences for unlimited medical treatment in hypothetical Alzheimer’s disease/dementia scenarios among community-dwelling adults. This cross-sectional study surveyed 163 community-dwelling adults (18+), using structured questionnaires to collect sociodemographic, health, and AdvDirs-related data. Key predictors included attitudes toward life-sustaining treatments, comfort discussing death, religious practices, and interest in quality-of-life information related to end-of-life care. The primary outcome was preference for unlimited medical treatment in hypothetical dementia scenarios. Bivariate and multivariate logistic analyses assessed associations, adjusting for covariates. In the dementia scenario, 26.9% of participants preferred unlimited medical treatment. This preference was strongly associated with a pre-existing attitude favoring life-sustaining treatments (OR = 4.24, 95% CI: 1.73 – 10.37, p = 0.002) and religious beliefs (OR = 5.68, 95% CI: 1.51–21.43, p = 0.01). Conversely, an interest in learning about quality of life at the end of life was negatively associated with preferring unlimited treatment (OR = 0.29, 95% CI: 0.09-0.89, p = 0.03). Our findings highlight the need to align advance care planning with individuals’ values, beliefs, and religious practices. Raising awareness of quality-of-life considerations in end-of-life care may lead to a shift in preference toward palliative care rather than aggressive treatment. Healthcare providers should discuss treatment trade-offs with cultural and religious sensitivity to support informed decision making.

